# Osteopathic manual therapy in fibromyalgia: a pilot study

**DOI:** 10.1590/1806-9282.20260646

**Published:** 2026-10-12

**Authors:** Aziz Dengiz, Elif Gur Kabul, Gulsum Tikac, Bilge Basakci Calik, Uğur Karasu

**Affiliations:** 1 Gaziantep Islam Science and Technology University, Faculty of Health Sciences, Department of Physiotherapy and Rehabilitation – Gaziantep, Türkiye.; 2 Usak University, Faculty of Health Sciences, Department of Physiotherapy and Rehabilitation – Uşak, Türkiye.; 3 Siirt University, Faculty of Health Sciences, Department of Physiotherapy and Rehabilitation – Siirt, Türkiye.; 4 Pamukkale University, Faculty of Physiotherapy and Rehabilitation, Department of Orthopedic Rehabilitation – Denizli, Türkiye.; 5 Pamukkale University, Faculty of Medicine, Department of Internal Medicine – Denizli, Türkiye.

**Keywords:** Fibromyalgia, Manipulative medicine, Osteopathic medicine, Physical therapy modalities, Exercise therapy

## Abstract

**OBJECTIVE:**

The aim of this pilot study was to evaluate the preliminary efficacy and feasibility of integrating Osteopathic Manual Therapy Techniques combined with Clinical Pilates on pain, symptom severity, and sleep quality in patients with fibromyalgia.

**METHODS:**

Twenty women with fibromyalgia were randomized into Osteopathic Manual Therapy Techniques+Clinical Pilates (n=11) and Clinical Pilates (n=9) groups. The protocol lasted 6 weeks (3 sessions/week). Osteopathic Manual Therapy Techniques included rib raising, suboccipital myofascial release, Sternocleidomastoid (SCM) mobilization, diaphragm/mediastinum techniques, and visceral mobilizations. Primary outcomes were the Widespread Pain Index, Symptom Severity Scale, and Pressure Pain Threshold. Sleep was assessed via the Pittsburgh Sleep Quality Index.

**RESULTS:**

Both groups showed significant intra-group improvements (p<0.05). Within-group analyses revealed that the Osteopathic Manual Therapy Techniques+Clinical Pilates group achieved significant improvements in Widespread Pain Index (p=0.003), Symptom Severity Scale (p=0.005), and Pittsburgh Sleep Quality Index (p=0.007). However, in the between-group analysis of change scores, the Osteopathic Manual Therapy Techniques+Clinical Pilates group demonstrated significantly superior improvements exclusively in sleep quality (Pittsburgh Sleep Quality Index, p=0.034) and maximal Pressure Pain Threshold at the trapezius (p=0.035) compared to the Clinical Pilates-only group, while between-group differences for Widespread Pain Index and Symptom Severity Scale did not reach statistical significance (p>0.05).

**CONCLUSION:**

Integrating Osteopathic Manual Therapy Techniques into exercise-based rehabilitation presents promising preliminary evidence for enhancing central sensitization symptoms and sleep quality in fibromyalgia patients, though larger trials are needed.

## INTRODUCTION

Fibromyalgia (FM) is a chronic syndrome characterized by widespread musculoskeletal pain accompanied by fatigue, sleep, memory, and mood issues^1^. FM is fundamentally characterized by central sensitization and a profound imbalance of the autonomic nervous system, where persistent sympathetic overactivity and suppressed parasympathetic function lead to abnormal pain processing. This autonomic dysregulation results in systemic vasoconstriction and subsequent ischemic events within the tissues, which are hypothesized to be the primary drivers of widespread musculoskeletal pain, fatigue, and hypersensitivity^2^. Osteopathic Manual Therapy (OMT) is hypothesized to address these mechanisms by employing specific manual techniques aimed at modulating autonomic tone—potentially downregulating the sympathetic "fight or flight" response and enhancing parasympathetic activity—while simultaneously improving local blood flow to reverse tissue ischemia and soothe the hypersensitive central nervous system^3^. The present study is important in terms of applying techniques that aim to affect the autonomic nervous system and measuring the effectiveness of these techniques.

While Clinical Pilates (CP) is effective, pain-related barriers often limit results. Osteopathic Manual Therapy Techniques (OMTT) aim to balance the autonomic nervous system^2^. We aimed to investigate the preliminary therapeutic benefits and clinical applicability of osteopathic manual techniques for pain and symptom management in FM patients, thereby providing feasibility data for larger, multi-center trials.

## METHODS

This study was approved by the Local Ethics Committee (Decision no: 09, Date: 05.30.2023) and performed in accordance with the Declaration of Helsinki. All participants provided written informed consent prior to inclusion.

In this randomized controlled clinical trial, conducted in accordance with the Consolidated Standards of Reporting Trials (CONSORT) guidelines, participants were assigned to either the Intervention Group (Osteopathic Manual Therapy Techniques+Clinical Pilates, OMTT+CP) or the Control Group (CP only) via a simple, unconstrained computer-generated randomization sequence (SPSS v.22.0) with a 1:1 allocation ratio.

The study included 28 women with FM. FM was diagnosed by the same rheumatologist. Following eight dropouts due to personal reasons in the OMTT+CP group (n=3) and hectic work schedules in the CP group (n=5), the remaining 20 women were included in the final analysis, as detailed in the CONSORT flow diagram (Figure 1). Inclusion criteria: (1) Female, aged 18–65 years; (2) Diagnosed with FM based on the 2016 American College of Rheumatology (ACR) criteria; (3) Stable medication dose for at least 6 months. Exclusion criteria: (1) Presence of neurological, orthopedic, cardiopulmonary, or inflammatory diseases; (2) Cognitive impairment; (3) Pregnancy; (4) Participation in regular exercise or rehabilitation programs for the 3 months in the preceding year.

**Figure 1:**
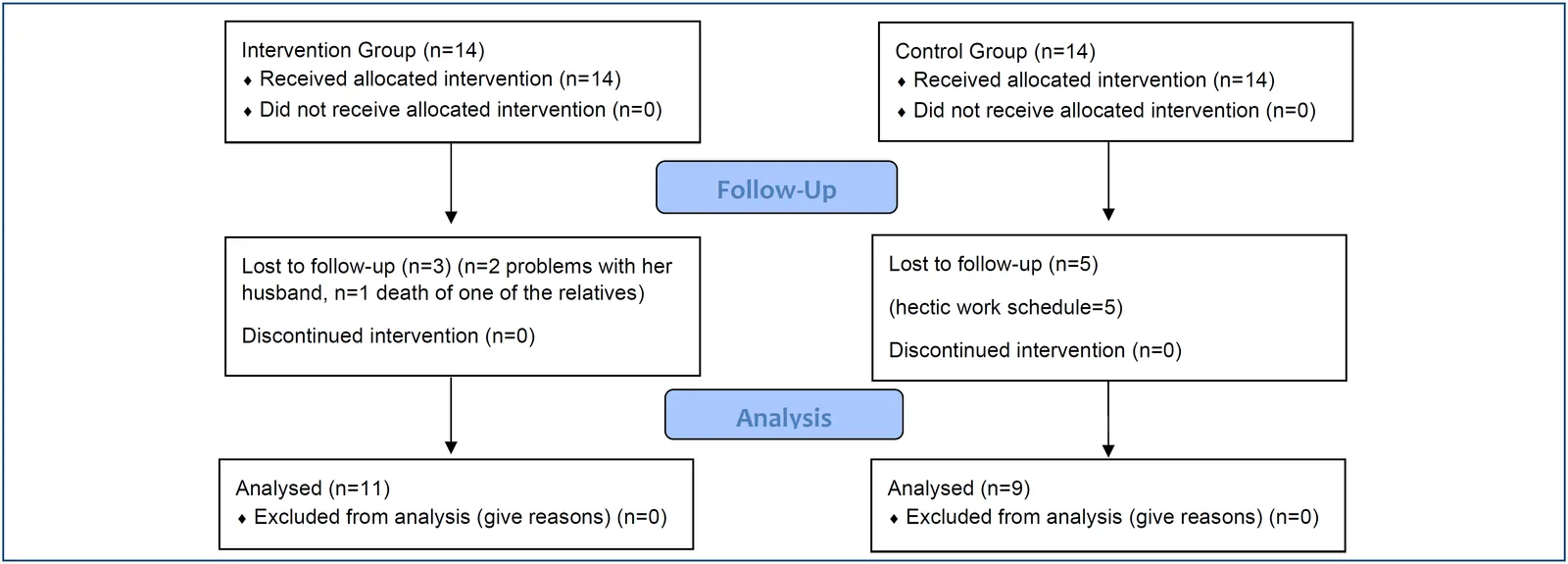
Consolidated Standards of Reporting Trials flow diagram of the study.

### Interventions

Both groups performed CP sessions three times weekly for 6 weeks.

Clinical Pilates (CP): CP sessions were conducted as group therapy under the supervision of a single physiotherapist. Prior to the intervention, participants were instructed in the five key elements of Pilates: lateral costal breathing, centering (focus), and the neutral alignment of the rib cage, shoulder girdle, and head/neck. Each 60-min session comprised a 10-min warm-up, 40 min of core-stabilization exercises, and a 10-min cool-down. Exercises initially consisted of 6–8 repetitions, with progression implemented at weeks 2 and 4 by increasing repetitions or adding resistance via elastic bands (e.g., advancing from yellow to red or green Theraband) based on individual tolerance.

Osteopathic Manual Therapy (OMTT): The intervention group received 30-min OMTT sessions prior to CP. All OMTT interventions were administered as a standardized protocol in every single session (three times weekly for 6 weeks, totaling 18 sessions). To eliminate inter-examiner variability, all sessions were delivered by a single, certified osteopathic practitioner/physiotherapist with over 5 years of clinical experience in manipulative medicine. The techniques were applied in a strict, uniform chronological sequence in accordance with a standardized protocol adapted from Nicholas and Nicholas. The specific clinical sequence and precise application durations were executed as follows:

1. Rib raising technique: Applied for approximately 2 min with the patient in the supine position while performing bilateral oscillation over the ribs.
2. Suboccipital myofascial technique: Applied for an average of 4–5 min in the supine position, utilizing finger semi-flexion between the occiput and axis until suboccipital relaxation was achieved, followed by light traction.
3. Bilateral SCM mobilization: Executed for an average of 2–3 min with anterior-posterior mobilization of the right and left SCM muscles in the supine position.
4. Mediastinum technique: Performed for an average of 8–10 repetitions with the patient in the side-lying position, applying synchronized multi-directional pressures to the sternum and thoracic region.
5. Diaphragm mobilization techniques: Administered via a three-step sequence (transverse costal mobilization for 1–2 min in supine with knees bent, respiratory-resisted manual tracking under the costal arch in supine, and manual costal border resistance in a sitting position).
6. Sacrum mobilization technique: Applied for 2 min with the patient in the prone position, delivering anterior-cranial oscillations at a rate of 150–180 beats per minute over the lower third of the sacrum.
7. Celiac and mesenteric ganglion oscillation technique: Performed for an average of 4 min in the supine position with knees flexed, applying manual oscillation therapy under the xiphoid process (celiac ganglion) and 2 cm caudally (mesenteric ganglion)^4^.

### Assessments

Evaluations were performed at baseline and after 6 weeks by a blinded researcher.

Primary outcomes: Pain intensity and symptom severity were measured using the Widespread Pain Index (WPI), Symptom Severity Scale (SSS), and Pressure Pain Threshold (PPT).

Secondary outcomes: Functional status (FIQ), sleep quality (PSQI), fatigue (MAF), and quality of life (SF-36) were assessed using validated Turkish versions of the respective scales. Exercise beliefs were evaluated with the Exercise Benefits/Barriers Scale (Exercise Beliefs Questionnaire-EBBS). Anxiety (BAI) and biopsychosocial status (Biopsychosocial Questionnaire [BETY-BQ]) were also recorded.

The WPI records widespread pain by questioning 19 specific regions (0–19 range). Symptom severity was measured by the SSS (0–12), which rates fatigue, sleep quality, and cognitive symptoms (0–3 points), and identifies the presence of headache, abdominal pain, and depression^5^. PPT were assessed at four bilateral sites (trapezius, lateral epicondyle, quadriceps femoris, and medial knee) using a digital algometer (JTECH Medical™ Commander Echo). The 1-cm^2^ rubber-tipped probe was applied perpendicularly to the skin, with pressure increased steadily until the participant reported the pain threshold. For each site, both maximal and mean force values (kg/cm^2^) were recorded^6^.

The FIQ, developed by Salaffi et al. to assess functional status in FM patients, comprises 10 items with a total score up to 100, where higher results indicate more severe impairment^7^. Sleep quality was evaluated using the PSQI, which scores seven components to provide a total range of 0–21, with higher values reflecting poorer sleep quality^8^. Fatigue was quantified via the 16-item MAF scale, measuring its severity and impact on daily activities^9^. Health-related quality of life was measured using the SF-36, where higher physical and mental component scores represent better overall well-being^10^. Additionally, the 43-item EBBS was used to determine exercise perceptions and barriers^11^, while the BAI assessed anxiety severity, with higher scores indicating increased anxiety levels^12^. Finally, the BETY-BQ evaluated the biopsychosocial status across 30 items, where higher scores signify lower health status^13^.

### Statistical analysis

Sample size calculation was performed via G*Power 3.1. To achieve 80% power with a 5% type-1 error rate (α=0.05) and an effect size of d=1.09^14^, a minimum of 24 subjects (12 per group) was required. To account for a potential drop-out rate during the 6-week follow-up, we intentionally over-recruited and randomized 28 participants (14 per group), exceeding the minimum requirement of 24 subjects. However, due to an unexpected attrition of eight participants during the follow-up period (reasons detailed in Figure 1), the final analysis was completed with 20 participants (11 intervention, 9 control).

Data were analyzed using International Business Machines Statistical Package for the Social Sciences Statistics v.22.0. Normality was checked via the Shapiro-Wilk test. Due to the small sample size (n=20) and the non-normal distribution of several clinical parameters, non-parametric statistical methods were applied. Non-normally distributed continuous variables are expressed as median (min–max). The Wilcoxon signed-rank and Mann-Whitney U tests were used for intra-group and inter-group comparisons, respectively. Between-group comparisons of therapeutic efficacy were performed using change scores (Delta=post - pre) via the Mann-Whitney U test. Although analysis of covariance is widely preferred to control for baseline variances in larger trials, it was not methodologically feasible in this study due to the violation of parametric assumptions (normality and sample size constraints) required for general linear models. Statistical significance was set at p<0.05.

## RESULTS

Twenty female patients with FM completed the study (Intervention: n=11; Control: n=9). The participant flow is detailed in the CONSORT diagram (Figure 1). Baseline demographic and disease-related characteristics showed no significant differences between the groups (Mann-Whitney U test, all p>0.05). Specifically, the median (min/max) age was 52 (38/62) years for the intervention group and 51.5 (30/63) years for the control group (p=0.469). Median height and weight were 1.56 m (1.52/1.66) and 74 kg (56/90) in the intervention group, compared to 1.60 m (1.55/1.67) and 74.5 kg (53/99) in the control group (p=0.731 and p=0.820, respectively). Furthermore, the median disease duration was 3.5 (1/11) years for the intervention group and 2 (0.5/22) years for the control group (p=0.360). Although slight baseline discrepancies existed in clinical parameters (such as disease duration and FIQ scores), these differences did not reach statistical significance, and subsequent inter-group evaluations were strictly performed using "change scores" (post-treatment minus baseline) to eliminate any potential confounding effects of minor baseline variances.

Following the treatment, intra-group analyses revealed that the intervention group (OMTT+CP) demonstrated significant improvements in WPI, SSS, overall sleep quality (PSQI), fatigue (MAF), and both the Physical and Mental components of the SF-36. However, no significant changes were observed in FIQ, BETY-BQ, or BAI scores within this group. Conversely, the control group showed significant post-treatment improvements only in MAF and BETY-BQ scores, with no significant changes in PPT values, WPI, SSS, PSQI, or SF-36 components (Table 1).

**Table 1:** Intragroup results of women with fibromyalgia.

Variables		Intervention group (n=11)				Control group (n=9)			
		Pre/median (min–max)	Post/median (min–max)	z	p	Pre/median (min–max)	Post/median (min–max)	z	p
Maximal PPT									
	m. trapezius	4.60 (3.50/8.10)	7.70 (4.80/12.70)		**0.003**	5.22 (2.90/7.10)	4.20 (0.80/8.10)		0.780
	Lateral epicondyle	4.15 (2.50/10.80)	6.85 (2.80/10.15)		**0.010**	3.15 (1.70/5.80)	3.55 (0.95/7.45)		0.470
	m. quadriceps femoris	8.10 (4.25/13.75)	11.30 (4.65/19.40)		**0.041**	6.22 (4.10/10)	5.52 (0.9/14.05)		0.432
	Medial knee	5.40 (3.20/8.70)	7.20 (2.60/14.85)		**0.013**	3.07 (1.10/9.45)	2.45 (0.55/9.45)		0.055
Mean PPT									
	m. trapezius	4.15 (3.15/7.90)	7.05 (3.80/11.05)		**0.004**	4.72 (2.70/6.50)	3.80 (0.70/7.25)		0.507
	lateral epicondyle	3.90 (2.15/9.05)	6.45 (2.45/9.35)		**0.017**	2.77 (1.45/5.45)	3.12 (0.75/6.05)		0.730
	m. quadriceps femoris	7.4 (4.05/11.85)	10.80 (4.45/18.50)		**0.033**	5.52 (3.60/8.70)	5.10 (0.75/12.15)		0.510
	Medial knee	5 (3.05/8.50)	6.90 (2.35/14.20)		0.068	2.50 (0.90/8.75)	2 (0.55/9.25)		0.124
WPI		16 (9–19)	11 (0–15)	-2.938	**0.003** *	12 (5–19)	11 (2–19)	-1.069	0.285
SSS		8 (6–11)	4 (0–10)	-2.807	**0.005** *	6.5 (4–12)	5.5 (1–10)	-1.511	0.131
FIQ		64.75 (43.52–89.10)	58.03 (31.61–79.80)	-0.800	0.424	54.31 (18.66–78.77)	41.12 (5.45–86.24)	-1.572	0.116
PSQI (total)		9 (4–15)	6 (2–11)	-2.712	**0.007** *	7 (2–13)	7 (3–12)	-0.816	0.414
MAF		36.24 (20.54–44.72)	26.47 (12.50–40.50)	-2.756	**0.006** *	26.16 (12.27–47.72)	15.85 (9.77–33.68)	-1.992	**0.046** *
SF-36 physical		139.55 (62.50–237.50)	180.45 (82.50–320.00)	-2.194	**0.028** *	201.07 (40.0–327.5)	285.36 (200.00–355)	-1.761	0.078
SF-36 mental		160.99 (97.00–259.80)	215.34 (115.50–306.70)	-2.293	**0.022** *	229.54 (77.5–335.5)	278.37 (193.00–358.00)	-0.135	0.893
BAI		24 (7–43)	23 (7–46)	-0.119	0.906	18 (3–31)	12 (6–19)	0.000	1.000
BETTY total		56 (28–76)	55 (32–73)	-0.059	0.953	43 (21–77)	22 (10–35)	-2.371	**0.018** *
EBBS—benefits		70 (61–78)	69 (53–78)	-0.280	0.779	72 (61–78)	72 (61–78)	-0.447	0.655
EBBS—barriers		37 (17–54)	37 (26–48)	-0.059	0.953	30 (12–49)	22 (14–32)	-1.577	0.115

PPT: Pressure Pain Threshold, m: musculus, WPI: Widespread Pain Index, SSS: Symptom Severity Scale, FIQ: Fibromyalgia Impact Questionnaire, PSQI: Pittsburgh Sleep Quality Index, MAF: Multidimensional Assessment of Fatigue, BAI: Beck Anxiety Inventory, FM: Fibromyalgia, BETTY: Biopsychosocial Questionnaire, EBBS: Exercise Benefits/Barriers Scale,

* p<0.05. Wilcoxon Paired-Sample Test. The bold values indicates statistical significance (p<0.05).

In inter-group comparisons of the change scores, the intervention group exhibited significantly greater reductions in sleep quality (PSQI, p=0.034) compared to the control group. Regarding pressure pain thresholds (PPTs), a statistically significant between-group difference was observed only for the maximal PPT at the trapezius muscle (p=0.035). In contrast, the between-group differences for WPI (p=0.389) and SSS (p=0.210) change scores did not reach statistical significance (p>0.05), despite the significant intra-group improvements observed within the intervention cohort. Furthermore, the mean PPT change score for the trapezius demonstrated only a trend toward statistical significance (p=0.088), and no significant differences were found at the other anatomical sites (Table 2).

**Table 2:** Between-group comparison of change scores (Δ=post–pre) in women with fibromyalgia.

Variables		Intervention group Δ Median (min–max)	Control group Δ Median (min–max)	Z	p
PPT max					
	m. trapezius	2.20 (0.20–8.25)	0.10 (-4.25 to 1.35)	-2.112	**0.035** *
	lat. epicondyle	2.35 (-1.70 to 6.05)	0.37 (-2.35 to 3.35)	-1.106	0.269
	m. QF	2.75 (-1.65 to 11.30)	-0.02 (-5.35 to 4.05)	-0.402	0.687
	medial knee	1.25 (-0.60 to 9.45)	-0.55 (-3.75 to 0.85)	-1.358	0.175
PPT mean					
	m. trapezius	1.55 (-0.55 to 6.90)	-0.07 (-3.80 to 1.85)	-1.709	0.088
	lat. epicondyle	2.15 (-1.50 to 6.35)	0.35 (-2.35 to 2.55)	-1.206	0.228
	m. QF	2.90 (-1.55 to 11.00)	0.07 (-5.15 to 3.45)	-0.654	0.513
	medial knee	0.45 (-1.60 to 9.20)	-0.40 (-3.40 to 0.65)	-0.805	0.421
WPI		-4 (-13 to -1)	0 (-11 to 7)	-0.862	0.389
SSS		-3 (-9 to 0)	-2 (-4 to 3)	-1.253	0.21
FIQ		8 (-18.78 to 44.68)	10 (-4.20 to 41.09)	-0.804	0.421
BETTY total		0 (-20 to 15)	12 (1–20)	-1.617	0.106
EBBS benefits		1 (-10 to 12)	0 (-4 to 2)	-0.240	0.810
EBBS barriers		0 (-15 to 17)	5 (-2 to 19)	-0.770	0.441
PSQI		3 (-1 to 8)	0 (-1 to 2)	-2.123	**0.034** *
MAF		10 (-1.40 to 18.41)	12 (-1.91 to 32.50)	0.000	1.000
SF-36 physical		-41 (-110 to 15)	-60 (-117.5 to 10)	-0.738	0.461
SF-36 mental		-54 (-123 to 33)	-23 (-148.67 to 77)	-0.794	0.427
BAI		0 (-10 to 23)	0 (-7.07 to 7.07)	-0.171	0.864

m. QF: Musculus Quadriceps Femoris; lat. epicondyle: lateral epicondyle; PPT: Pressure Pain Threshold; WPI: Widespread Pain Index; SSS: Symptom Severity Scale; FIQ: Fibromyalgia Impact Questionnaire; MAF: Multidimensional Assessment of Fatigue; BAI: Beck Anxiety Inventory; BETTY: Biopsychosocial Questionnaire; EBBS: Exercise Benefits/Barriers Scale; PSQI: Pittsburgh Sleep Quality Index. Data are presented as median (minimum–maximum) change scores (Δ=post–pre), Between-group comparisons were performed using the Mann-Whitney U test,

* p<0.05. The bold values indicates statistical significance (p<0.05).

## DISCUSSION

The primary finding of this pilot study is that while CP alone improves FM symptoms, a multi-modal approach combining CP with OMTT shows promising trends and preliminary superiority in reducing pain and improving sleep quality compared to CP alone. Specifically, the intervention group demonstrated significant improvements in the number of painful areas, PPTs, and sleep quality compared to the control group. OMTT significantly boosts the efficacy of structured exercise.

Current international guidelines consistently identify exercise as the first-line non-pharmacological intervention for FM^15^. Among various exercise modalities, CP is highly recommended for its safety and efficacy in reducing pain, tenderness, and functional limitations. A recent systematic review and meta-analysis by Schaffer et al. confirmed that Pilates significantly improves pain and disability in FM patients^16^. In our study, the significant improvement in symptoms within the control group aligns with these recent findings, confirming that CP remains a robust therapeutic tool. However, the additional gains in the intervention group highlight that exercise alone may not fully address the complex autonomic and neuroendocrine dysfunctions inherent in FM.

FM is fundamentally characterized by central sensitization and a disrupted autonomic nervous system (ANS)^17^. Patients typically exhibit a state of "sympathovagal imbalance," marked by sympathetic hyperactivity and reduced parasympathetic tone^18^. OMTT is thought to target these specific pathways to help restore homeostasis. In our protocol, rib raising techniques (T1–T8) and ganglion mobilizations were utilized with the therapeutic intent of downregulating sympathetic tone. Theoretically, increased sympathetic activity leads to regional vasoconstriction and muscle hypoperfusion, which is a primary driver of localized tender points^19^. By potentially enhancing local circulation and oxygenation through these techniques might have contributed to the significant increase in PPT levels observed in our intervention group.

Simultaneously, we focused on facilitating parasympathetic activation via suboccipital myofascial release and sacrum mobilization. A recent meta-analysis by Cerritelli et al. demonstrated that OMTT induces a predominant parasympathetic shift, effectively modulating the ANS and increasing vagal tone^20^. Literature suggests that manual stimulation of fascial tissues may activate free nerve endings that communicate with the insular cortex and hypothalamus, potentially triggering a systemic parasympathetic response^21^. This "bottom-up" modulation is essential for patients with FM, who often show altered central pain processing.

It is worth noting that while the between-group analysis revealed a significant improvement in the maximal PPT of the trapezius muscle, the mean PPT for the same muscle reached only a trend toward significance. This divergence can be methodologically attributed to the localized nature of our pilot sample size and the inherent symptomatic volatility of FM patients. Maximal PPT may respond more rapidly to acute localized therapeutic interventions like suboccipital release and rib raising. Conversely, mean PPT scores are more susceptible to intra-session variability and baseline pain fluctuations, potentially diminishing statistical power in small cohorts.

Our results are strongly supported by recent high-quality trials. Arienti et al. conducted a randomized controlled trial demonstrating that OMTT significantly improves pain intensity and functional status in women with FM^22^. Similarly, Dal Farra et al. concluded that OMT sessions predominantly based on myofascial release play a therapeutic role in rapidly improving functional status and quality of life^23^.

Our findings contrast with older studies, such as Coste et al., who found no significant benefit of OMT over sham treatment^24^. We attribute this discrepancy to our treatment frequency and multi-modal design. While that study utilized OMT once weekly as a standalone, joint-focused therapy, our study integrated OMT with CP three times weekly, emphasizing soft tissue mobility. This supports the growing modern consensus that OMT is most effective as an adjunct therapy to exercise rather than a mono-therapy^25^.

The marked improvement in sleep quality (PSQI) in the OMTT group is one of our most vital findings. FM-related sleep disturbances are often resistant to standard exercise alone. By potentially encouraging a shift toward parasympathetic tone, OMTT facilitates a state of physiological relaxation, thereby improving sleep onset and overall sleep quality^20^.

An intriguing finding in our study was that despite significant improvements in pain distribution (WPI) and symptom severity (SSS) within the intervention group, these changes did not translate into a statistically significant reduction in overall FIQ scores. This apparent discrepancy can be attributed to several factors. First, the FIQ is a comprehensive instrument that evaluates long-term functional status, activities of daily living, and work difficulties over the past week. While OMTT successfully downregulates acute sympathetic hyperreactivity and rapidly modulates localized pain thresholds (PPT), altering a patient's deeply ingrained chronic functional limitations and perceived work difficulty likely requires a longer intervention period than 6 weeks. Second, as a pilot study with a small sample size, the final analysis was likely underpowered to detect smaller clinical changes in multi-dimensional scales like the FIQ, potentially increasing the risk of a Type II error. Nevertheless, the visual trend toward improvement in FIQ scores within the OMTT+CP group provides a promising foundation for future long-term trials.

To our knowledge, this is one of the first studies to specifically investigate the synergistic effects of OMTT and CP in FM within the context of recent neurobiological understandings. Furthermore, the pattern of statistical significance observed between intra-group and inter-group analyses warrants cautious interpretation. While the control group demonstrated significant intra-group improvements in fatigue (MAF) and biopsychosocial status (BETY-BQ)—confirming the well-established benefits of CP on general well-being—the between-group analysis revealed that the synergistic addition of OMTT specifically drove superior outcomes in localized mechanical pain thresholds (maximal PPT at the trapezius) and sleep quality (PSQI). This highlights that while exercise lays a foundational recovery for fatigue and biopsychosocial status, OMTT acts as a powerful targeted accelerator for central sensitization and autonomic sleep-wake regulation. The lack of significant between-group differences in MAF or BETY-BQ indicates that both interventions carry valuable, yet distinct, therapeutic weights.

The primary limitation of this study is that we did not objectively measure physiological autonomic parameters (e.g., heart rate variability, electrodermal activity) or local blood flow changes to directly confirm the mechanisms of action of OMTT. Therefore, our physiological interpretations regarding sympathetic downregulation and parasympathetic activation remain theoretical and speculative, based on indirect behavioral and symptomatic improvements. Additionally, the lack of biochemical markers (e.g., substance P or cortisol levels) limits our ability to objectively map neuroendocrine adaptations.

A key limitation is the small final sample size. Although initial randomization met the power threshold (n=28), dropouts due to personal reasons reduced the analyzed sample to 20. Consequently, the study may be underpowered, increasing Type II error risks or overestimating effect sizes. Furthermore, post-attrition unequal group sizes (n=11 vs. n=9) led to minor, non-significant baseline clinical differences. Although delta change scores minimized their impact, baseline imbalances might have subtly influenced outcomes. Thus, these pilot findings require cautious interpretation and validation through larger, fully powered trials employing restricted block randomization to ensure group equivalence.

Another significant limitation of this study is the lack of blinding for both the participants and the therapist delivering the interventions. Due to the nature of manual therapy and physical exercises, full double-blinding is methodologically challenging. Consequently, this lack of blinding may have introduced performance and expectancy biases, particularly regarding subjective, self-reported outcomes such as pain distribution (WPI), symptom severity (SSS), and sleep quality (PSQI). Participants receiving a more complex, multi-modal intervention (OMTT+CP) might have harbored higher expectations of clinical benefit compared to the control group. To mitigate this potential bias, the outcome assessments were strictly conducted by an independent, fully blinded researcher. Furthermore, we included an objective physical metric—PPT via digital algometry—to balance the subjective nature of the questionnaires and to provide a more resilient clinical evaluation.

In conclusion, combining OMTT with CP may yields enhanced clinical outcomes in pain reduction, PPTs, and sleep quality compared to Pilates alone within this pilot sample. Based on these preliminary findings, future multidisciplinary FM rehabilitation programs could consider exploring the integration of OMTT to address autonomic dysfunctions comprehensively, pending confirmation from larger, fully-powered trials.

## CONCLUSION

In conclusion, this pilot study provides encouraging preliminary evidence that osteopathic manual treatment techniques may potentially enhance the effectiveness of exercise in reducing pain and increasing the PPT in individuals with FM. As a preliminary investigation, these findings demonstrate the feasibility of the protocol but cannot be considered definitive. Robust, larger-scale randomized controlled trials with long-term follow-ups are strictly required to verify these clinical trends and establish standard guidelines.

## Data Availability

The datasets generated and/or analyzed during the current study are available from the corresponding author upon reasonable request.
